# Dysbiosis of Gut Microbiota in Patients With Acute Myocardial Infarction

**DOI:** 10.3389/fmicb.2021.680101

**Published:** 2021-07-05

**Authors:** Ying Han, Zhaowei Gong, Guizhi Sun, Jing Xu, Changlu Qi, Weiju Sun, Huijie Jiang, Peigang Cao, Hong Ju

**Affiliations:** ^1^Department of Cardiovascular, The Fourth Affiliated Hospital of Harbin Medical University, Harbin, China; ^2^College of Bioinformatics Science and Technology, Harbin Medical University, Harbin, China; ^3^Department of Cardiovascular, The First Affiliated Hospital of Harbin Medical University, Harbin, China; ^4^Department of Radiology, The Second Affiliated Hospital of Harbin Medical University, Harbin, China; ^5^Department of Cardiology, Heilongjiang Province Land Reclamation Headquarters General Hospital, Harbin, China; ^6^Department of Information Engineering, Heilongjiang Biological Science and Technology Career Academy, Harbin, China

**Keywords:** acute myocardial infarction, gut microflora, 16S rRNA gene, patients, dysbiosis

## Abstract

Acute myocardial infarction (AMI) continues as the main cause of morbidity and mortality worldwide. Interestingly, emerging evidence highlights the role of gut microbiota in regulating the pathogenesis of coronary heart disease, but few studies have systematically assessed the alterations and influence of gut microbiota in AMI patients. As one approach to address this deficiency, in this study the composition of fecal microflora was determined from Chinese AMI patients and links between gut microflora and clinical features and functional pathways of AMI were assessed. Fecal samples from 30 AMI patients and 30 healthy controls were collected to identify the gut microbiota composition and the alterations using bacterial 16S rRNA gene sequencing. We found that gut microflora in AMI patients contained a lower abundance of the phylum *Firmicutes* and a slightly higher abundance of the phylum *Bacteroidetes* compared to the healthy controls. Chao1 (*P* = 0.0472) and PD-whole-tree (*P* = 0.0426) indices were significantly lower in the AMI versus control group. The AMI group was characterized by higher levels of the genera *Megasphaera*, *Butyricimonas*, *Acidaminococcus*, and *Desulfovibrio*, and lower levels of *Tyzzerella 3*, *Dialister*, *[Eubacterium] ventriosum group*, *Pseudobutyrivibrio*, and *Lachnospiraceae ND3007 group* as compared to that in the healthy controls (*P* < 0.05). The common metabolites of these genera are mostly short-chain fatty acids, which reveals that the gut flora is most likely to affect the occurrence and development of AMI through the short-chain fatty acid pathway. In addition, our results provide the first evidence revealing remarkable differences in fecal microflora among subgroups of AMI patients, including the STEMI vs. NSTEMI, IRA-LAD vs. IRA-Non-LAD and Multiple (≥2 coronary stenosis) vs. Single coronary stenosis groups. Several gut microflora were also correlated with clinically significant characteristics of AMI patients, including LVEDD, LVEF, serum TnI and NT-proBNP, Syntax score, counts of leukocytes, neutrophils and monocytes, and fasting serum glucose levels. Taken together, the data generated enables the prediction of several functional pathways as based on the fecal microfloral composition of AMI patients. Such information may enhance our comprehension of AMI pathogenesis.

## Introduction

Despite timely reperfusion through primary percutaneous coronary intervention, acute myocardial infarction (AMI) is still the leading cause of morbidity and mortality worldwide (Nwokocha et al., 2017; Khan et al., 2020; Qi et al., 2021). It is really important to further reduce myocardial infarct size and preserve cardiac function, in order to reduce risk of death and prevent onset of heart failure. Therefore, it is necessary to thoroughly understand its pathogenic mechanism and find new therapeutic targets. Accumulating evidence reveals the role of gut microbiota in regulating the pathogenesis of coronary heart disease (CHD) (Cui et al., 2017; Liu et al., 2019, 2020; Moraru et al., 2019; Heianza et al., 2020; Marzullo et al., 2020), including the obvious association between the gut microbiota and the severity of AMI in rats (Lam et al., 2012, 2016; McCafferty et al., 2012).

There are a vast array of microbes in human gut, collectively referred to as the microbiota, which is a complex community. The metabolic activities and interactions with the immune system of gut microbiota are not limited the gut itself (Sonnenburg and Backhed, 2016), but also involve a variety of immune-mediated diseases and metabolic diseases such as diabetes, obesity, digestive system diseases, asthma, arthritis, cancers, and cardiovascular disease (Vieira et al., 2014; Sonnenburg and Backhed, 2016; Grigoryan et al., 2019; Ingham et al., 2019; Kolodziejczyk et al., 2019; Alemao et al., 2020; Morel et al., 2020; Yang et al., 2021). The abundance of *Enterobacteriaceae* and *Streptococcus* spp. were reported to be higher in patients with atherosclerotic cardiovascular disease compared to the healthy controls in a metagenome-wide association study (Jie et al., 2017). Koren et al. (2011) revealed that *Chryseomonas*, *Veillonella*, and *Streptococcus* exsited in AS plaque samples, and several bacterial flora in the intestine are the same as atherosclerotic plaques. Furthermore, they were related to the cholesterol levels (Koren et al., 2011). Trimethylamine-*N*-oxide (TMAO), a metabolite of gut microbiota, can partially promote the formation of atherosclerosis by promoting the formation of macrophage foam cells (Koeth et al., 2013; Tang et al., 2013). According to the study of Emoto et al. (2016) the alterations of gut microbiota were linked to the incidence of coronary artery disease.

Published studies on the role of the microbiome in coronary heart disease have investigated most patients with stable coronary heart disease using a cross-sectional design. However, there are few clinically meaningful prospective studies of the microbiome in patients with AMI. Therefore, in this study, fecal samples from AMI patients and healthy controls were collected, variable regions of gut bacterial 16S rRNA were amplified, and DNA library was constructed. The data of this study may provide detailed information on variations of gut microbial composition and its impacts on AMI.

## Materials and Methods

### Study Participants

Between April and August of 2020, we recruited 30 AMI in-hospital patients and 30 asymptomatic controls receiving routine physical examinations for this study. AMI patients were recruited from the First and Fourth Affiliated Hospitals of Harbin Medical University while the healthy controls were recruited from the Fourth Affiliated Hospital of Harbin Medical University. AMI diagnosis was based on the World Health Organization definition and the third universal definition of myocardial infarction and consisted of patients with symptoms of ischemia, cardiac laboratory biomarker data, electrocardiogram results and invasive coronary angiograms or coronary CT angiography (Mendis et al., 2011; Thygesen et al., 2012). The criteria for the healthy controls included no ischemic symptoms, normal electrocardiogram and coronary stenosis of <25% as assessed by invasive coronary angiograms or coronary CT angiography. The exclusion criteria consisted of subjects that: (i) received antacids, probiotics, antibiotics, or antimicrobial agents within 30 days before sample collection, (ii) had an organic disease of the digestive system, and (iii) had gastrointestinal surgery. All participants experienced a normal lifestyle prior to admission, including typical Chinese diets based on carbohydrates versus high-fat diets and participated in routine levels of general physical activity (e.g., housework and walking). However, activities of AMI patients were restricted following admission. All participants (or their direct relatives) gave written informed consent, and the First Affiliated Hospital of Harbin Medical University and the Fourth Affiliated Hospital of Harbin Medical University approved all study protocols.

### Sample Collection and DNA Extraction

Fresh fecal samples (each 2–5 g) were collected from all the participants under the hospital diet, then transferred into sterile collecting pipes and frozen at –80°C immediately. The associated clinical data were collected simultaneously. The bacterial DNA was extracted from the fecal samples using the TIANamp Bacteria DNA kit (Tiangen, Beijing, China) according to the manufacturer’s instructions.

### 16S rRNA Gene V3–V4 Region Sequencing

DNA extracted from each sample was used as a template, and the V3–V4 region of the 16S rRNA gene was amplified using PCR. PCR amplification, sequencing of the PCR amplicons and quality control of raw data were performed. The purified products were mixed in equal proportions for sequencing.

### Sequencing Data Analysis

First, we evaluated the quality of sequencing data using the Fast-QC software^1^. Second, clean Data were obtained for subsequent analysis after removing the Chimera Sequence using QIIME2^2^. Third, Operational taxonomic units (OTUs) were delineated at the cutoff of 97% also using QIIME2, and then the sequencing results were compared and analyzed to obtain the family and genus annotations of OTUs based on the Silva database^3^. Fourth, α- and β-diversity analyses were performed using QIIME2. Shannon–Wiener diversity index, Simpson diversity index, the observed OTUs, PD (phylogenetic diversity)-whole-tree and Chao1 index were evaluated. A normalized OTU abundance table was used for the β-diversity analysis, including principal coordinate analysis (PCoA) based on weighted UniFrac, and unweighted UniFrac distances. Next, Lefse analysis was performed to clarify the dominant bacteria. LEfSe is a software for discovering high-dimensional biomarkers and revealing genome characteristics. LEfSe uses linear discriminant analysis (LDA) (Yang et al., 2020) to estimate the impact of the abundance of each component (species) on the difference effect. Finally, the gene function of the sample was inferred based on the species composition obtained by sequencing, and the functional difference between different groups was analyzed using PICRUSt^4^. Subsequently, the Welch’s *t*-test method of two groups was performed using the STAMP software to filter the parts with *P*-value > 0.05, and Heatmap Plot, PCA plot, and Extended error bar graphs were drawn to reveal significant differences in species abundance between different samples. Based on the data obtained by sequencing, we performed the differential taxonomy expression analysis using limma algorithm for screening, and the differential screening criteria are: LogFC > 0.585 or <–0.585, *P*-value < 0.05.

## Results

### Baseline Characteristics

A summary of the baseline characteristics of all the participants is presented in Table 1. AMI patients were characterized as consisting of a greater number of males, worsened cardiac functions, larger left ventricular end diastolic diameter (LVEDD), increased serum Troponin I (TnI) and NT-pro B-type natriuretic peptide (NT-proBNP) levels, increased numbers of leukocytes, neutrophils, and monocytes, increased fasting blood glucose levels and an increased prevalence of comorbidity with hypertension. In addition, among the patients enrolled in this study, including 15 ST-elevation myocardiol infarction (STEMI) and 15 non-ST elevation myocardiol infarction (NSTEMI), 19 experienced left anterior descending coronary (LAD) stenosis as the infarction related artery (IRA) and 21 had two or more coronary artery stenosis.

**TABLE 1:** Baseline characteristics of the study participants.

Variables	AMI patients(*n* = 30)	Healthy controls(n = 30)	*P*-value
Age, years	62.6(9.02)	60.0(9.64)	0.2915
Sex, male	18(60%)	10(33%)	0.0390
BMI(kg/m^2^)	25.4(3.33)	24.9(3.08)	0.5685
STEMI(%)	15(50%)	—	—
IRA (LAD)	19(63%)	—	—
≥2 coronary stenosis	21(70%)	—	—
Syntax score	18.1(5.95)	—	—
NYHA class (I/II/III/IV)	13/7/5/5	—	—
Hypertension	22(73%)	11(37%)	0.0038
Diabetes	9(30%)	5(16.7%)	0.2291
Atrial fibrillation	2(6.7%)	0(0)	0.1555
Smoking	13(43.3%)	6(20%)	0.0533
**Echocardiogaraphic parameters**			
LVEDD, mm	48.6(5.09)	43.7(3.99)	<0.0001
LVEF, %	51.7(8.78)	63.2(4.65)	<0.0001
SV, ml	52.2(12.06)	58.9(11.94)	0.0383
E/e’	64(60-68)	60(58–65)	<0.0001
**Laboratory parameters**			
TnI, ng/dL	27.6(0.10–226.9)	0.012(0–0.048)	0.0120
NT-proBNP, pg/mL	2652.1(113–26259)	124.0(25–258)	<0.0001
Leukocyte, 10^9^/L	8.6(2.73)	6.7(1.74)	0.0019
Neutrophils, 10^9^/L	6.0(2.62)	4.0(1.30)	0.0006
Lymphocytes, 10^9^/L	2.1(1.17)	2.1(0.65)	0.9859
Monocyte, 10^9^/L	0.7(0.31)	0.4(0.14)	<0.0001
Hemoglobin, g/L	133.6(34.77)	140.8(17.04)	0.3140
BUN, mg/dl	6.2(2.42)	5.5(1.81)	0.1714
Serum creatinine, mg/dl	76.1(35.38)	67.4(18.35)	0.2415
Fast glucose	6.6(2.21)	5.2(1.30)	0.0061
Cholesterol	4.6(1.33)	5.0(0.90)	0.1339
Triglycerides	1.8(0.76)	1.9(1.66)	0.5977
HDL-C	1.0(0.59)	43(58.11)	0.3584
LDL-C	2.6(0.87)	2.9(0.80)	0.1643
Uric acid	340.5(95.17)	316.9(73.03)	0.2853

*Results are presented as median (with standard error or upper and lower quartiles) or % where appropriate.BMI, body mass index; STEMI, ST-elevation myocardiol infarction; IRA, Infarction related artery; LAD, left anterior descending coronary; NYHA, New York Heart Association; LVEDD, left ventricular end diastolic diameter; LVEF, Left ventricular ejection fraction; TnI, Troponin I; NT-proBNP, NT-pro B-type natriuretic peptide; HDL-C, high density lipoprotein-cholesterol; LDL-C, low density lipoprotein-cholesterol.*

### Species Classification

**FIGURE 1:**
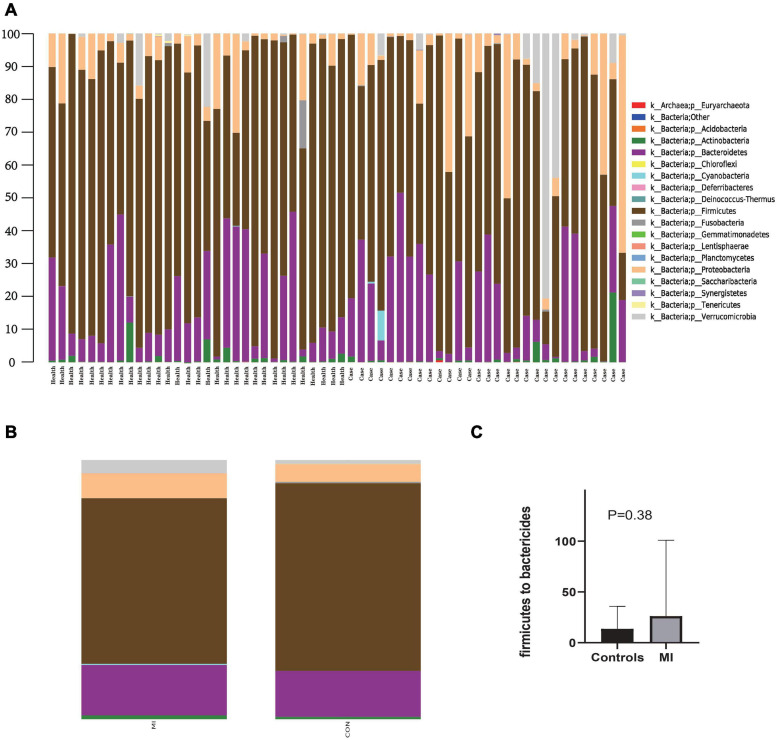
The distribution of relative abundance of top 20 at the phylum level. **(A)** Shows the distribution of relative abundance of top 20 in each fecal sample. **(B)** Shows the distribution of relative abundance of top 20 in AMI group and the healthy control group. MI, AMI group; CON, the healthy control group. **(C)** Shows the *Firmicutes* to *Bactericides* ratio in AMI group and the healthy control group.

Figure 1 contains a summary of the overall distribution of relative abundance of the top 20 phyla in each fecal sample (Figure 1A), as well as those found within each of the two groups (Figure 1B). Sequencing analysis revealed that the gut microbiota of the two groups were mainly contained within four phyla, *Bacteroidetes*, *Firmicutes*, *Proteobacteria*, and *Verrucomicrobia* (Figure 1B). The phylum with the highest abundance of reads in AMI patients was *Firmicutes*, accounting for 63.8% in total, versus that of an abundance of 72.4% in the controls (Figure 1B). The second greatest abundance in AMI patients was the phylum *Bacteroidetes*, accounting for 19.5% in total as compared with 17.7% in the controls (Figure 1B). Compared with the healthy control group, there was a rising trend but no significance of *Firmicutes* to *Bactericides* ratio in the patients with AMI (Figure 1C). Overall, there was a greater abundance in AMI versus controls for bacteria belonging to the phyla *Actinobacteria* (1.5 vs. 0.9%), *Cyanobacteria* (0.4 vs. 0.0%), *Proteobacteria* (9.6 vs. 6.8%), and *Verrucomicrobia* (5.0 vs. 1.5%). While a greater abundance was observed in controls versus AMI patients for bacteria belonging to the phyla *Fusobacteria* (0.55 vs. 0.1%) and *Tenericutes* (0.1 vs. 0.0%).

At the genus level, the microflora of AMI patients was characterized by lower levels of *Faecalibacterium*, *Roseburia*, *Tyzzerella 3*, [*Eubacterium*] *ventriosum group*, [*Eubacterium*] *rectale group*, *Ruminococcaceae NK4A214 group*, *Ruminococcaceae UCG-013*, *Ruminococcaceae UCG-014*, *Ruminococcus 1*, *Ruminococcus 2*, *Ruminococcaceae uncultured*, *Erysipelotrichaceae UCG-003*, *Megamonas*, *Fusobacterium*, and *Parasutterella*, and higher levels of *Bifidobacterium*, *Butyricimonas*, *Parabacteroides*, *Chloroplast; Other; Other; Other*, *Lysinibacillus*, *Lactobacillus*, *Christensenellaceae R-7 group*, *Subdoligranulum*, [*Eubacterium*] *coprostanoligenes group*, *Phascolarctobacterium*, *Megasphaera*, *Veillonella*, *Klebsiella*, and *Akkermansia* (Supplementary Figure 1).

### Analysis of α and β Diversity Index

**FIGURE 2:**
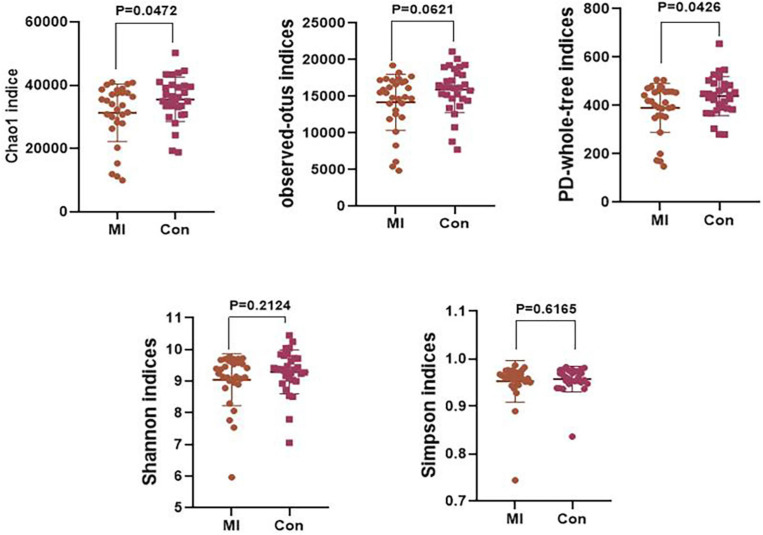
The level of α-diversity indices of the gut microflora between the AMI group and the healthy control group. MI, AMI group; CON, the healthy control group.

An α diversity analysis was performed and the resultant chao1, observed-outs, PD-whole-tree, Shannon–Wiener, and Simpson curves as based on the species annotation information obtained by sequencing analysis are presented in Supplementary Figure 2. The chao1 (*P* = 0.0472) and PD-whole-tree (*P* = 0.0426) indices were significantly decreased in the AMI versus control group (Figure 2). However, no statistically significant differences were obtained in the Shannon and Simpson indices. Taxonomic compositions of the metagenomic populations of gut microflora samples from AMI patients were compared with those from the healthy control group using the Principal Coordinate Analysis (PCoA). Differences in β-diversity as based on unweighted and weighted UniFrac values between the AMI and control group are shown in Supplementary Figure 2. The results of this analysis indicates that the fecal microbial structure of the AMI group differs from that of the healthy control group with regard to the presence of OTU.

### Difference Expression Analysis Between the AMI and Control Groups

**FIGURE 3:**
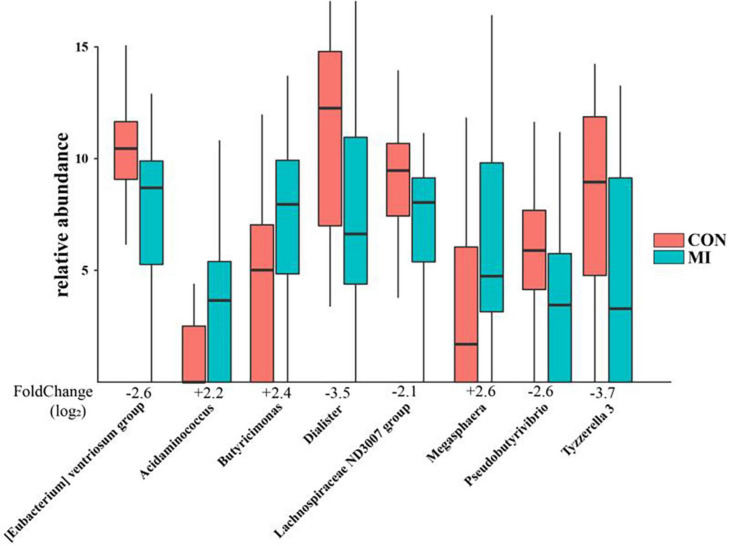
The remarkable results of differential taxonomy expression analysis using limma algorithm between the AMI and healthy control group. MI, AMI group; CON, the healthy control group.

A differential taxonomy expression analysis was performed using limma algorithms. When focusing on differences at the genus level, our results revealed a remarkable difference with 50 generus in fecal microflora between the AMI and healthy control group. Among these changes, the increases in *Megasphaera*, *Butyricimonas*, *Acidaminococcus*, and *Desulfovibrio*, and decreases in *Tyzzerella 3*, *Dialister*, *[Eubacterium] ventriosum group*, *Pseudobutyrivibrio*, and *Lachnospiraceae ND3007 group* were the most notable features (Figure 3).

We also searched in the gutMDisoeder database (Cheng et al., 2020) whether the above gut microbes associated with AMI have the same pattern of changes as other diseases, or the opposite pattern of change with intervention measures, and the results were showed in Tables 2, 3. The increase of genera *Megasphaera* were reported to be related to Parkinson’s disease and metabolic syndrome, and the reduced abundance was observed by giving Vitamin D intervention. The increase of genera *Desulfovibrio* were reported to be associated with multiple sclerosis and gestational diabetes, and the reduced abundance was observed by giving *N*-acetylcysteine or dextran sulfate sodium intervention. The decrease of genera *Dialister* were reported to be related to asthma, thyroiditis, and type 1 diabetes mellitus, and the increased abundance was observed by giving polydextrose or soluble corn fiber intervention.

**TABLE 2:** The gut microbes associated with AMI have the same pattern of change as other diseases searched in the gutMDisoeder database.

Gut microbe	Alteration	Disorder
Megasphaera	Up	Parkinson’s disease
Megasphaera	Up	Metabolic syndrome
Butyricimonas	Up	Infectious diarrhea
Acidaminococcus	Up	Parkinson’s disease
Acidaminococcus	Up	Idiopathic calcium stone
Acidaminococcus	Up	Colorectal cancer
Acidaminococcus	Up	Inflammatory bowel disease
Desulfovibrio	Up	Microscopic colitis
Desulfovibrio	Up	Multiple sclerosis
Desulfovibrio	Up	Gestational diabetes
Desulfovibrio	Up	Familial adenomatous polyposis
Desulfovibrio	Up	Inflammatory bowel disease
Desulfovibrio	Up	Hepatic encephalopathy
Desulfovibrio	Up	Infectious diarrhea
Desulfovibrio	Up	Human immunodeficiency virus infectious disease
Dialister	Down	Asthma
Dialister	Down	Thyroiditis
Dialister	Down	Familial Mediterranean fever
Dialister	Down	Type 1 diabetes mellitus
Dialister	Down	Henoch-Schoenlein purpura
Dialister	Down	Spinal cord injury
Pseudobutyrivibrio	Down	Acute myeloid leukemia
Pseudobutyrivibrio	Down	Spinal cord injury
Lachnospiraceae ND3007 group	Down	Idiopathic calcium stone

**TABLE 3:** The gut microbes associated with AMI have the opposite pattern of change with interventions searched in the gutMDisoeder database.

Intervention	Alteration	Gut microbe
Vitamin D	Down	Megasphaera
Bifico	Down	Desulfovibrio
JinQi Jiangtang	Down	Desulfovibrio
Perilla oil	Down	Desulfovibrio
*N*-acetylcysteine	Down	Desulfovibrio
Dextran sulfate sodium	Down	Desulfovibrio
Polydextrose	Up	Dialister
Soluble corn fiber	Up	Dialister

### Differences Between the Subgroups in Patients With AMI

An analysis of the differences in the composition were performed among subgroups of AMI patients including, STEMI vs. NSTEMI, IRA-LAD vs. IRA-Non-LAD and Multiple (≥2 coronary stenosis) vs. Single coronary stenosis groups. Remarkable differences in fecal microflora were found among these subgroups.

**FIGURE 4:**
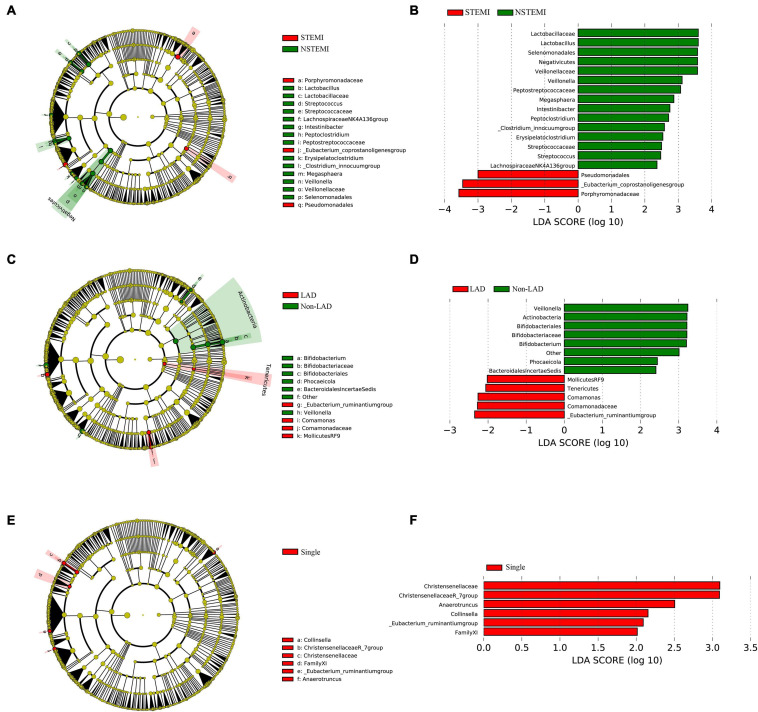
The difference expression of gut microflora between the subgroups in patients with AMI. **(A,B)** Shows the different abundances of gut microflora between the STEMI group and the NSTEMI group; **(C,D)** shows the different abundances of gut microflora in the patients with LAD as the IRA compared to the IRA-Non-LADs; **(E,F)** shows the different abundances of gut microflora in the Single coronary stenosis group than in the Multiple (≥2 coronary stenosis) group. STEMI, ST-elevation myocardiol infarction; NSTEMI, Non ST-elevation myocardiol infarction; IRA, Infarction related artery; LAD, left anterior descending coronary.

Mean abundances of the genera *Pseudomonadales*, *Eubacterium-coprostanoligenes group*, and *Porphyromonadaceae* were greater in the STEMI vs. NSTEMI group, while a significantly greater abundance of the genera *Streptococcaceae*, *Lachnospiraceae NK4A136 group*, *Lactobacillus*, *Intestinibacter*, *Veillonella*, *Streptococcus*, *Peptostreptococcaceae*, *Erysipelatoclostridium*, *Veillonellaceae*, *Megasphaera*, *Lactobacillaceae*, *Peptoclostridium*, and *[Clostridium]innocuum group* were found in the NSTEMI vs. STEMI group (Figures 4A,B).

With regard to the IRA-LAD vs. IRA-Non-LAD subgroup, our results suggested a significantly greater abundance of the genera *[Eubacterium]ruminantium group*, *Comamonadaceae*, *Comamonas*, and the bacteria belonging to the order *MollicutesRF9*, as well as the bacteria belonging to the *Tenericutes* phyla in patients with LAD as the IRA vs. the IRA-Non-LAD subgroup. Whereas mean abundances of the genera *Veillonella*, *Bifidobacteriaceae*, *Bifidobacterium*, *Phocaeicola*, and others belonging to the *Lachnospiraceae* family, bacteria belonging to the *BacteroidalesIncertaeSedis* family and bacteria belonging to the *Actinobacteria* class were greater in the IRA-Non-LAD vs. IRA-LAD group (Figures 4C,D).

When comparing the Multiple (≥2 coronary stenosis) vs. Single coronary stenosis groups, a significantly greater abundance of the genera *[Eubacterium]ruminantium group*, *Christensenellaceae*, *Christensenellaceae R-7 group*, *Collinsella*, and *Anaerotruncus*, as well as the bacteria belonging to *FamilyXI* were observed in the Single coronary stenosis group (Figures 4E,F).

### Correlations Between the Clinical Characteristics and the AMI Microflora

**FIGURE 5:**
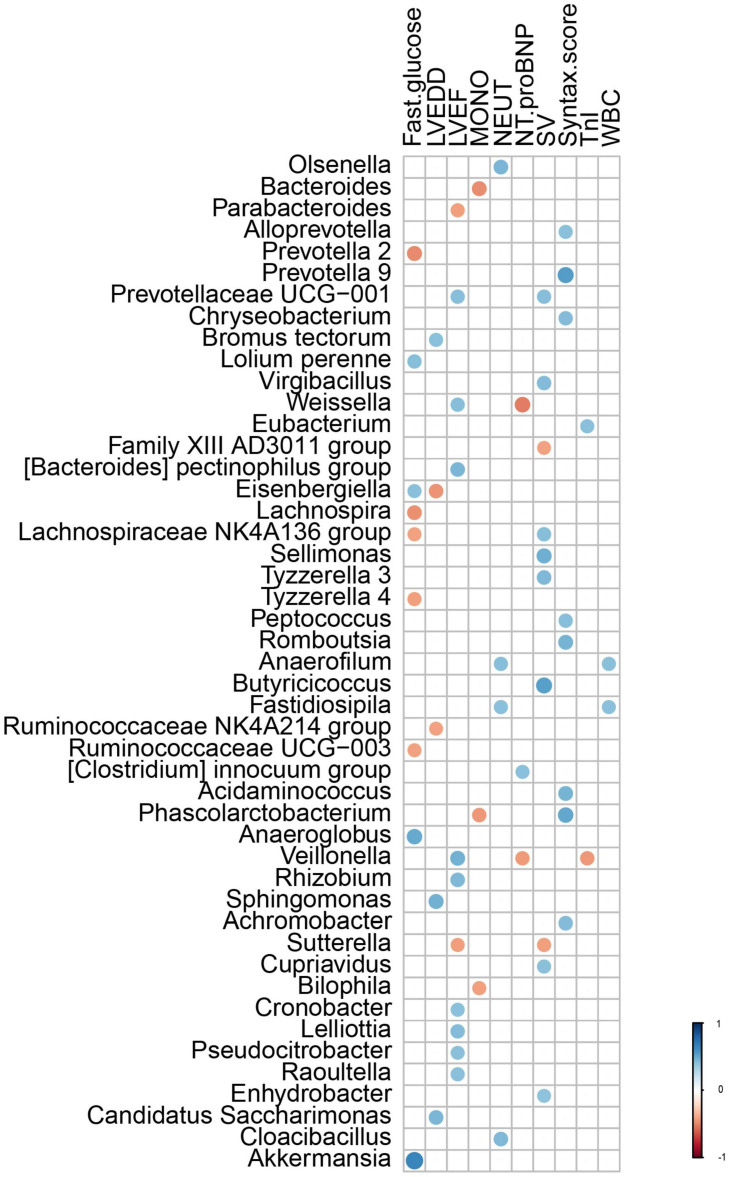
The correlation between the gut microflora and the clinical characteristics with different significance in AMI patients. LVEDD, left ventricular end diastolic diameter; LVEF, left ventricular ejection fraction; TnI, Troponin I; NT-proBNP, NT-pro B-type natriuretic peptide; WBC, white blood cell; NEUT, neutrophils; LYMPH, lymphocytes; MONO, Monocyte.

Correlations were performed between the composition of gut microflora and significant clinical characteristics within AMI patients. Results of these analyses revealed that the genera *Bromus tectorum*, *Sphingomonas*, and *Candidatus Saccharimonas* were positively correlated with LVEDD, while the genera *Eisenbergiella* and *Ruminococcaceae NK4A214 group* were negatively correlated with LVEDD. The genera *Prevotellaceae UCG-001*, *Weissella*, *[Bacteroides] pectinophilus group*, *Veillonella*, *Rhizobium*, *Cronobacter*, *Lelliottia*, *Pseudocitrobacter*, and *Raoultella* were positively correlated with left ventricular ejection fraction (LVEF), while the genera *Parabacteroides* and *Sutterella* were negatively correlated with LVEF. The genus *Eubacterium* was positively correlated with levels of TnI, while the genus *Veillonella* was negatively correlated with TnI levels. The genus *[Clostridium] innocuum group* was positively correlated with levels of NT-pro-BNP, while the genera *Weissella* and *Veillonella* were negatively correlated with NT-pro-BNP levels. The genera *Alloprevotella*, *Prevotalla9*, *Chryseobacterium*, *Peptococcus*, *Romboutsia*, *Acidaminococcus*, *Phascolarctobacterium*, and *Achromobacter* were positively correlated with Syntax scores. The genera *Anaerofilum* and *Fastidiosipila* were positively correlated with leukocyte and neutrophil counts, *Olsenella* and *Cloacibacillus* were positively correlated with counts of neutrophils and the genera *Bacteroides*, *Phascolarctobacterium*, and *Bilophila* were negatively correlated with counts of monocytes. The genera *Eisenbergiella*, *Lolium perenne*, *Anaeroglobus*, and *Akkermansia* were positively correlated with fasting serum glucose levels, while *Lachnospiraceae NK4A136 group*, *Prevotella 2*, *Lachnospira*, *Tyzzerella 4*, and *Ruminococcaceae UCG-003* were negatively correlated with these fasting glucose levels (Figure 5).

### Predictive Functional Analysis

**FIGURE 6:**
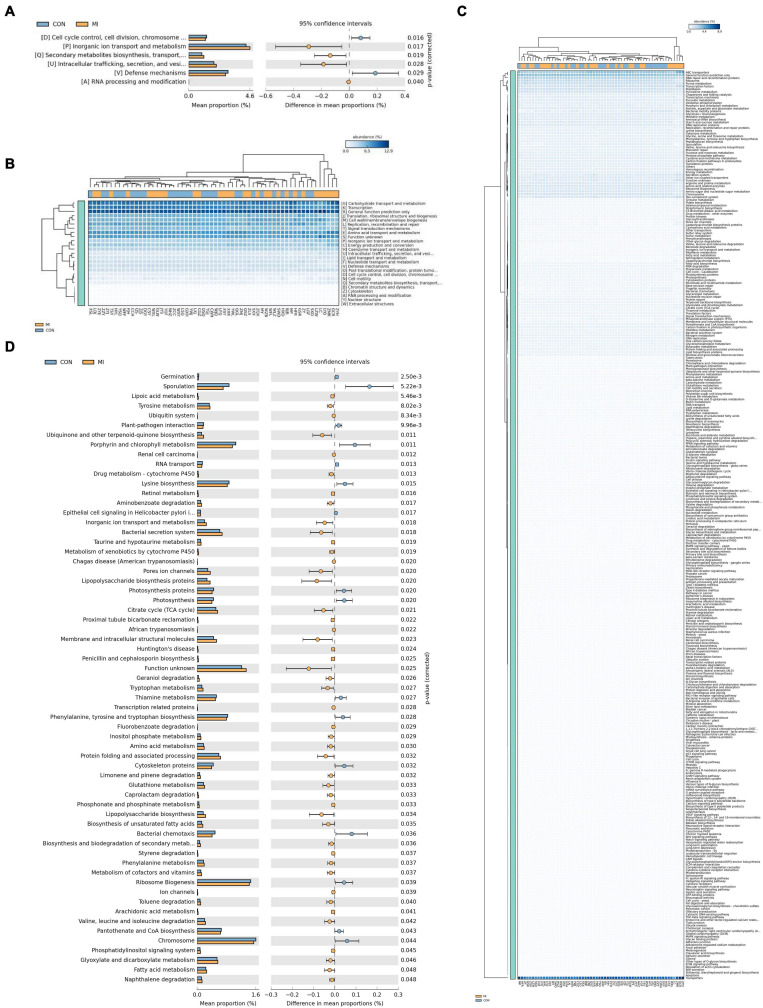
The results of PICRUst based on closed-reference OTU to predict the abundances of functional categories COG orthologs (COs) and KEGG orthologs (KOs). **(A,B)** Shows the COs with significantly different abundances in the fecal microbiome between the AMI group and healthy control group; **(C,D)** shows the KOs with significantly different abundances in the fecal microbiome between the AMI group and healthy control group. MI, AMI group; CON, the healthy control group.

PICRUst, as based on closed-reference OTU, was applied to predict abundances of the functional category COG orthologs (COs) and KEGG orthologs (KOs). Some of these COs and KOs demonstrated significantly different abundances in fecal microbiomes between the AMI and healthy control group (*P* < 0.05; Figure 6). Results from the COG database indicated that, inorganic ion transport and metabolism functioning, intracellular trafficking, secretion/vesicular transport, secondary metabolite biosynthesis, transport/catabolism and RNA processing/modification were all significantly increased in the AMI group. In contrast, defense mechanisms and cell cycle control functions, cell division and chromosome partitioning were significantly increased in the healthy control group (*P* < 0.05 for both groups; Figures 6A,B). However, at the level of KEGG pathways, we found significant increases in the AMI versus control group for the following processes: functioning of lipopolysaccharide biosynthesis proteins, membrane and intracellular structural molecules, biosynthesis of ubiquinone and other terpenoid-quinones, bacterial secretion, inorganic ion transport/metabolism, ionic pore channels, lipoic acid metabolism, tyrosine metabolism, the ubiquitin system, drug metabolism of cytochrome P450, aminobenzoate degradation, the citrate (TCA) cycle, tryptophan metabolism, geraniol degradation, protein folding and associated processing, amino acid metabolism, inositol phosphate metabolism, glutathione metabolism, limonene and pinene degradation, lipopolysaccharide biosynthesis, unsaturated fatty acid biosynthesis, valine, leucine and isoleucine degradation, biosynthesis/biodegradation of secondary metabolites, and fatty acid metabolism (*P* < 0.05; Figures 6C,D).

## Discussion

In the current study, fecal samples from 30 AMI patients and 30 healthy controls were collected to identify the composition and alterations in gut microbiota between these two groups as determined using bacterial 16S rRNA gene sequencing. Our results demonstrated a number of notable differences in gut microbial composition between these AMI patients and healthy controls. The composition of gut microflora was significantly correlated with clinical characteristics in AMI patients for such parameters as LVEDD, LVEF, serum TnI and NT-proBNP, Syntax scores, counts of leukocytes, neutrophils and monocytes, and fasting glucose levels. Moreover, significant differences in abundances of fecal microbiomes between the AMI and control group were obtained for some COs and KOs. We also analyzed differences in expressions among various subgroups of AMI patients. From this analysis we provide the first evidence indicating that remarkable differences in fecal microflora are present between the STEMI vs. NSTEMI, IRA-LAD vs. IRA-Non-LAD, and the Multiple (≥2 coronary stenosis) vs. Single coronary stenosis subgroups.

The gut microbiota, as the “second genome” of humans, is affected by a host of genes. Although the host genotype plays a decisive role in the composition and structure of the gut microbiota, the effect of diet cannot be ignored. Mounting evidence suggests that diet represents one of the most important factors influencing the composition and structure of gut microbiota (Cotillard et al., 2013), with changes in diet having the capacity to exert beneficial or harmful effects upon the composition of gut microbiota of the host. For example, it has been reported that a high-fat diet can damage the intestinal microbial environment and lead to microbiome dysregulation by reducing the amount of available carbohydrates in the colon, as well as increasing the level of intestinal oxygen stress and its own secondary metabolites (Ge et al., 2020; Li et al., 2020). In our study, all participants were from the same region, experienced a normal/routine lifestyle and had similar nutritional patterns, including typical Chinese diets based on carbohydrates versus high-fat diets. Furthermore, all the participants, including the healthy controls, were subjected to the hospital diet to minimize potential confounding effects of dietary differences on the microflora. We found that significant differences were present between AMI patients and healthy controls with regard to the fecal microbiome, suggesting the existence of a link between gut microflora dysbacteriosis and AMI. At the phylum level, *Firmicutes* and *Bacteroidetes*, the two most abundant phyla inhabiting the intestinal tract, are closely associated with environmental conditions and can be either beneficial or problematic to human and animal health. In addition, *Bacteroidetes* were reported to be implicated in immune regulation including activation of inflammation and autoimmune diseases (Carr et al., 2002; Gibiino et al., 2018; Nadia and Ramana, 2020). Our results suggest that the abundance of *Firmicutes* is decreased, while *Bacteroidetes* are slightly elevated in AMI patients. These findings are consistent with results obtained in an animal model of isoproterenol-induced AMI (Sun et al., 2019), but differ from results as obtained from fecal samples of patients with coronary heart disease (Kelly et al., 2016; Cui et al., 2017; Wang et al., 2018). One possible explanation for these results is that AMI, as a type of coronary heart disease with abrupt exacerbation and high mortality, has a unique pathophysiological process, including acute thrombosis, myocardial necrosis, inflammation, activation of the neuroendocrine system, and ventricular remodeling, may produce changes in gut microbiota. In addition, differences in gut environments may affect the abundance and composition of gut microbiota.

Microbiota diversity has emerged as a new biomarker of health (Shanahan, 2010; Cheng, 2019; Aponte et al., 2020; Ma et al., 2020). Loss of gut flora biodiversity is associated with various diseases, including active inflammatory bowel disease, childhood autism and recurrent *Clostridium difficile* associated diarrhea (Ott et al., 2004; Chang et al., 2008). In contrast, increased microbiota diversity is associated with enhanced health in the elderly (Claesson et al., 2012). Our current results show that the chao1 and PD-whole-tree indices of α-diversity were significantly decreased in the AMI group, revealing that the community richness of gut microbiota significantly decreased in the AMI patients. However, no statistically significant differences were obtained in other indices, including the Shannon index, results which may be attributable to the relatively small sample size.

Further differential taxonomy expression analyses using limma algorithms enabled us to focus on differences at the genus level. The AMI group was characterized by higher levels of *Megasphaera*, *Butyricimonas*, *Acidaminococcus*, and *Desulfovibrio*, and lower levels of *Tyzzerella 3*, *Dialister*, *[Eubacterium] ventriosum group*, *Pseudobutyrivibrio*, and *Lachnospiraceae ND3007 group* as compared with that observed in the healthy controls. *Megasphaera* belongs to the strictly anaerobic gram-negative cocci, which is involved in fermenting fructose and lactic acid with some short-chain fatty acids (SCFA) such as acetic acid and propionic acid main products as the main metabolites. *Butyricimonas*, which converts glucose into butyric and isobutyric acid, can also generate other types of SCFA such as acetic, propionic and succinic acid. These SCFA are an important source of energy for intestinal mucosal cells, contribute to the construction/repair of the intestinal mucosal barrier and resist oxidative stress (Sakamoto et al., 2014). *Desulfovibrio* can convert sulfates in food into sulfides, with hydrogen sulfide exerting a dual effect on gastrointestinal function through its capacity to either protect the gastrointestinal tract or participate in intestinal injury (Zhang-Sun et al., 2015). These bacterial genera have specific metabolic functions, and in individuals rich in these bacteria, lower levels of trimethylamine *N*-oxide (TMAO) are present, which is a major factor influencing cardiovascular diseases. *Acidaminococcus* is an anaerobic diplococcus that can use amino acids as their only source of energy for growth, which also belongs to the strictly anaerobic gram-negative cocci and produce acetic acid and butyric acid as metabolites. *Dialister* is one of the most representative types of intestinal flora associated with irritable bowel syndrome, and is believed to be correlated with dialister enrichment (Lopetuso et al., 2018). The main products are lactic acid, acetic acid and formic acid. *Tyzzerella* has been reported to be richly abundant in the patients with a high risk for cardiovascular diseases (Kelly et al., 2016; Xu et al., 2019), with the products of glucose fermentation include formic acid, lactic acid, acetic acid, ethanol, CO_2_, and H_2_. Only a few studies are available on the bacteria *[Eubacterium] ventriosum group* and *Lachnospiraceae ND3007 group*, accordingly, the physiological and pathological effects of most of these bacteria remain unclear. But it is certain that the main metabolites of these two genus are also SCFA. Even though the OTUs were assigned to the same genus, their functions may be distinct, as functions of bacteria are strain specific (Zhao, 2013). However, the common metabolites of the above-mentioned significantly different bacterial genera are mostly SCFA, which reveals that according to our results, the gut flora is most likely to affect the occurrence and development of AMI through the SCFA pathway. We also searched in the gutMDisoeder database and found that the pattern of changes in the gut microbes associated with AMI were the same as that of some other diseases such as Parkinson’s disease, metabolic syndrome, multiple sclerosis, gestational diabetes and type 1 diabetes mellitus, or opposite to the pattern of changes in some interventions such as Vitamin D, *N*-acetylcysteine, dextran sulfate sodium, polydextrose, and soluble corn fiber. Most of these diseases are related to metabolism, obesity, immunity, and inflammation, and these factors also play an important role in the occurrence and development of AMI. Therefore, to a certain extent, it supports the hypothesis that change of gut flora participates in the pathophysiological process of AMI. Furthermore, some interventions such as Vitamin D and *N*-acetylcysteine might be useful for the treatment of AMI. These results will provide new direction for the role of intestinal flora in the pathophysiological process of AMI, as well as new targets for the treatment of AMI.

Our data also suggest that the composition of the gut microflora was significantly correlated with clinical characteristics of AMI patients, including LVEDD, LVEF, serum TnI and NT-proBNP, Syntax scores, WBC counts (neutrophils and monocytes), and fasting glucose levels. In specific, the correlations obtained indicated that gut microflora were associated with a greater incidence of LVEDD and lower incidence of LVEF suggesting that the gut microflora was involved with impaired cardiac function and left ventricular remodeling in AMI patients. And, the severity of AMI was characterized by serum levels of TnI and NT-proBNP. Furthermore, these indicators were significantly related to the prognosis of AMI patients, insinuating a role for gut microflora in the outcome of AMI patients. Among all gut microflora, the genera *Weissella* and *Veillonella* were positively correlated with LVEF and negatively correlated with levels of NT-pro-BNP indicating their role in cardiac functions. Similar to *Lactobacillus*, the genus *Weissella* was found to have a probiotic potential as a type of lactic acid bacteria (Anandharaj et al., 2015) with lactic acid and short-chain fatty acids as the metabolites, while the genus *Veillonella* has been reported to decompose lactic acid to produce propionic acid and promote metabolism (Scheiman et al., 2019), which also suggests that short-chain fatty acids play an important role in AMI. A sterile inflammatory environment is considered to be of paramount importance for AMI and ischemia/reperfusion injury development (Braunwald, 2015; Han et al., 2020), and is accompanied with elevated counts of leukocytes, neutrophils and monocytes. Like that as reported in other studies (Tang and Hazen, 2014; Yamashita et al., 2015; Amoroso et al., 2020; Wang et al., 2020), we found that gut microflora was related to immunity. The genera *Anaerofilum* and *Fastidiosipila* are both positively correlated with leukocyte and neutrophil counts, suggesting that they might be closely related to the sterile inflammatory conditions required for the pathophysiological process of AMI. In addition, gut microflora have been widely reported to be related with glucose and lipid metabolism (Qin et al., 2012; Karlsson et al., 2013; Fu et al., 2015), which is similar to our current results showing that gut microflora was related to fasting glucose levels.

To our knowledge, this is the first study that has examined differences in gut microflora among subgroups of AMI patients, especially the IRA-LAD vs. IRA-Non-LAD and Multiple (≥2 coronary stenosis) vs. Single coronary stenosis groups. Our results revealed that remarkable differences in fecal microflora were present between the STEMI vs. NSTEMI, IRA-LAD vs. IRA-Non-LAD and Multiple (≥2 coronary stenosis) vs. Single coronary stenosis groups. These findings not only indicate that gut microflora play an important role in the severity of AMI, but are also related to LAD occlusion and multiple coronary stenosis. Among the remarkable differences observed in fecal microflora of the subgroups, we found that nearly all of the bacterial genera belonged to the *Firmicutes* phyla in the NSTEMI and Single coronary stenosis groups, while most of the bacterial genera belonged to the *Proteobacteria* phyla in the STEMI and IRA-LAD groups. We were not able to further identify any direct correlations or mechanisms. In this way, the abundance of specific fecal microflora may possess the potential for prediction of pathophysiological and clinical characteristics of AMI.

Based on closed-reference OUT, PICRUst was applied for predictive functional analysis. Several functional pathways, including inorganic ion transport and metabolism, secondary metabolite biosynthesis, transport, catabolism, protein folding and associated processing, amino acid metabolism, inositol phosphate metabolism and the Citrate (TCA) cycle have been identified. These functional pathways play an important role in such pathophysiological processes of AMI including myocardial necrosis, activation of acute inflammation, reperfusion injury and myocardial post-infarction repair. Therefore, these pathways can serve as a means to further predict the gut microflora that may contribute to AMI development, and it is probable due to its metabolite SCFA according to our results. SCFAs not only have the function of oxidative energy supply, but also have important functions such as maintaining water and electrolyte balance, anti-inflammatory, regulating immunity, regulating oxidative stress, anti-tumor and regulating gene expression. As the gut microbial ecosystem, which is considered as the largest endocrine organ of the body, can produce a variety of biologically active compounds that can be transported through the circulation and distributed to the distant parts of the host body, a plethora of basic biological and pathophysiological processes can impact the host (Tremaroli and Backhed, 2012). Therefore, long-term follow-up functional studies are urgently needed to reveal the specific bacteria that may contribute to the processes of AMI progression.

There are limitations with this current study. The relatively small sample sizes and lack of age/sex matched subjects for the AMI and control groups is a factor warranting consideration. The samples were only collected at a single time point, which precludes any assessments as to whether microbiota may fluctuate in patients with AMI during their treatment. Finally, multiple omics data will be required to further clarify the correlations between gut microbiota and AMI, as well as to establish the mechanisms through which gut microbiota affect the pathophysiological processes of AMI. In fact, it may be that multi-factorial processes are involved, which remains a subject for further investigation. Such determinations will likely need to be performed in animal models.

## Conclusion

In conclusion, the present study suggested that the composition and the diversity of gut microflora were different between the AMI patients and healthy controls. Some fecal microflora were also found to be closely related to AMI clinical characteristics, as well as the alterations in the gut microbial community in different subgroups of AMI patients. Moreover, our results predicted several functional pathways based on the fecal microfloral information from AMI patients, which may enhance our comprehension of AMI pathogenesis. Overall, the process of AMI progression is dynamic and complicated, and modulation of the gut microbiota composition may represent a promising diagnostic biomarker or therapeutic target. In conclusion, the present results reveal that the composition and the diversity of gut microflora markedly differ between AMI patients and healthy controls. Since the common metabolites of the significantly different bacterial genera are mostly short-chain fatty acids, the gut flora is most likely to affect the occurrence and development of AMI through the short-chain fatty acid pathway. Some fecal microflora were found to be positively correlated with AMI clinical characteristics and distinct alterations in the gut microbial community were present within different subgroups of AMI patients. Moreover, our results show that predictions of several functional pathways can be generated as based on the fecal microfloral data from AMI patients. Such information may enhance our comprehension of AMI pathogenesis. Overall, the process of AMI progression is dynamic and complicated, and modulation of the gut microbiota composition may represent a promising diagnostic biomarker or therapeutic target.

## Data Availability Statement

The data presented in the study are deposited in the SRA database repository accession number PRJNA733305.

## Ethics Statement

The studies involving human participants were reviewed and approved by the First Affiliated Hospital of Harbin Medical University and the Fourth Affiliated Hospital of Harbin Medical University approved all study protocols. The patients/participants provided their written informed consent to participate in this study.

## Author Contributions

HJi, PC, and HJu conceived and designed the experiments. JX, GS, and WS analyzed the data. CQ drew the pictures. YH and ZG wrote this manuscript. All the authors read and approved the final manuscript.

## Conflict of Interest

The authors declare that the research was conducted in the absence of any commercial or financial relationships that could be construed as a potential conflict of interest.

## Footnotes

1 http://www.bioinformatics.babraham.ac.uk/projects/fastqc/

2 http://qiime.org/

3 https://www.arb-silva.de/

4 https://picrust.github.io/picrust/index.html

## Supplementary Material

Supplementary Figure 1 The distribution of relative abundance at the genus level.

Supplementary Figure 2 α and β Diversity Index of the gut microflora.
