# Supplementary figures and images for: Dysbiosis of Gut Microbiota in Patients With Acute Myocardial Infarction
Source: Front Microbiol. 2021 Jul 5;12:680101. doi: 10.3389/fmicb.2021.680101 (PMC8290895; doi:10.3389/fmicb.2021.680101)

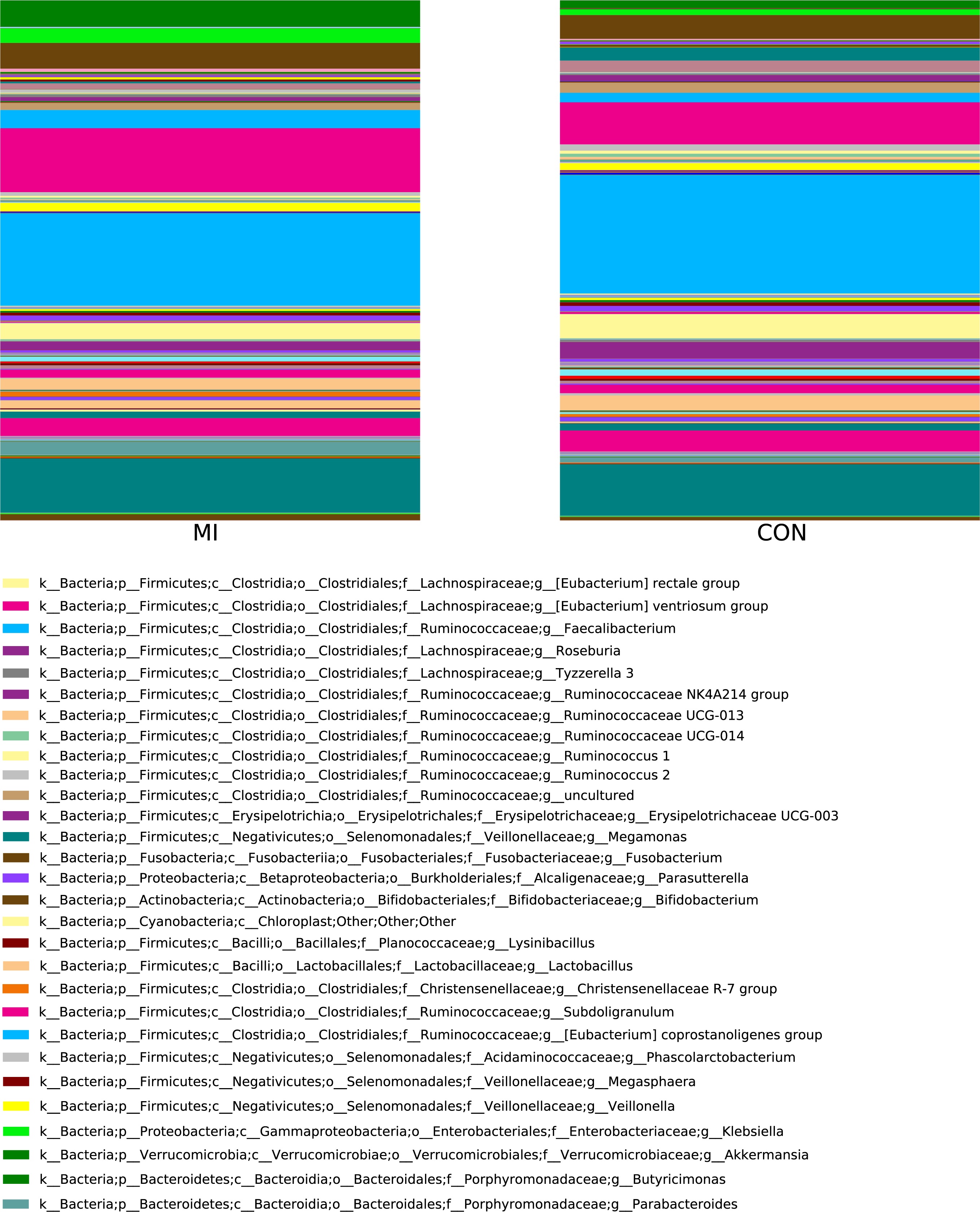

Supplement: Supplementary Figure 1 — The distribution of relative abundance at the genus level. [file Image_1.JPEG]

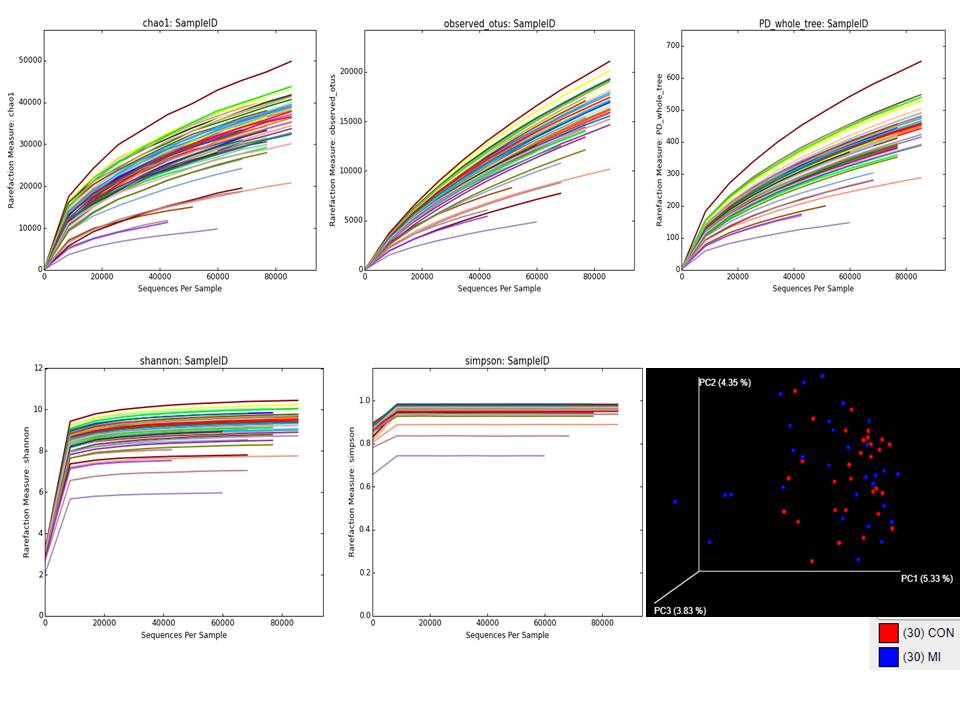

Supplement: Supplementary Figure 2 — α and β Diversity Index of the gut microflora. [file Image_2.JPEG]
